# Two or More Synchronous Combination of Noninvasive Test to Increase Accuracy of Liver Fibrosis in Chronic Hepatitis C

**DOI:** 10.5812/hepatmon.6229

**Published:** 2012-05-30

**Authors:** Roberto Testa

**Affiliations:** 1 Internal Medicine Gastroenterology Unit St Corona Hospital, Savona, Italy

**Keywords:** Liver Fibrosis, Hepatitis C, Chronic, Biopsy

Dear Editor,

The staging of liver fibrosis in chronic hepatitis C (CHC) is mandatory for both starting antiviral therapy and for starting a surveillance program for the early diagnosis of HCC. Considering that liver biopsy (LB) is “the best but not the gold standard” for the evaluation of fibrosis owed to possible limitations in the sample specimen and histological interpretation [1], the noninvasive evaluation of significant and severe fibrosis by means of direct or indirect biochemical and “ultrasound” fibrosis indexes is a highly complementary tool in the management of Chronic Hepatitis C (CHC). Some algorithms which are using a combination of direct and indirect fibrosis tests have been validated [2], but the interesting goal of the paper of Crisan et al. [3] was to combine a simple, inexpensive test with more complex noninvasive models (NITs) or Transient Elastography (TE). The gain in diagnostic accuracy of various combinations was about 10 %, and this could help to avoid a significant number of LBs. The main limitation of this study was the length of the LB sample which was below the limit of adequate staging and grading of liver disease, and thus may have limited the discriminant performance of NITs and particularly of TE. However, paradoxically the partial reliability of the histological staging, that is usual in clinical practice, highlighted the potential value of the NITs in fibrosis staging. In this context, it is likely that all NITs showed an underestimated ability to distinguish in both significant and severe fibrosis which is fundamentally depends on a histological bias. In fact, the diagnostic accuracy of the various NITs proved to be lower than in the literature data [4]. The combination of APRI or FIB4 in more complex models or in measurements of liver stiffness allows for the compensation of this histological gap. Particularly, in identifying significant fibrosis the combinations of APRI and FIB4 with Fibro meter showed a very high PPV which can safely allow us to avoid LB, while in discriminant severe fibrosis (practically cirrhosis) both APRI and FIB4 combined with Fibro test showed an NPV that may have delayed surveillance for HCC. In this study, APRI increased the diagnostic ability of TE to identify significant fibrosis (from 64.55 % to 79.78 %), and both APRI and FIB4 to identify severe fibrosis (from 79.66 to 85.71 and to 87.83, respectively). Considering the relatively high cost of TE, Fibro test and Fibro meter, the diagnostic gain allows us to improve the cost/benefit ratio of these more complex tests. This study shows how the diagnostic performance of an expensive test can be improved by combining it with an inexpensive test. Thus, a synchronous evaluation provides a more reliable tool to distinguish the CHC patients in order to identify fibrosis staging, likely reducing LB in clinical practice.
